# Evaluating the Implementation Quality of a Social and Emotional Learning Program: A Mixed Methods Approach

**DOI:** 10.3390/ijerph17093249

**Published:** 2020-05-07

**Authors:** Katherine Dowling, Margaret M. Barry

**Affiliations:** World Health Organization Collaborating Centre for Health Promotion, National University of Ireland Galway, University Road, H91TK33 Galway, Ireland; margaret.barry@nuigalway.ie

**Keywords:** social and emotional learning, school-based programs, implementation quality, mixed methods, adolescence, mental health and well-being

## Abstract

School-based social and emotional learning (SEL) programs have been shown to be effective in producing positive outcomes for adolescents. However, variability in implementation quality can have a negative impact on these program effects. The aim of this current study is to examine the variability in implementation quality for schools implementing the MindOut program and to identify factors that were likely to contribute to this variability. Employing a mixed methods approach, quantitative and qualitative implementation data were collected from teachers (n = 16) and students (n = 280) who participated in the MindOut program. Quantitative indicators were used to score schools’ implementation quality across four dimensions (dosage, adherence/fidelity, quality of delivery and participant responsiveness), and these were averaged to determine overall level of implementation (high/low). Qualitative data identified factors that contributed to implementation quality, and factors were then analyzed in accordance with the schools’ implementation level grouping. Findings indicated that variability in implementation quality existed both between and within schools. A total of eight schools were assigned as high implementers and another eight as low implementers. Influencing factors were categorized into five themes: (i) program factors, (ii) participant factors, (iii) teacher factors, (iv) school contextual factors, and (v) organizational capacity factors. Several differences between high and low implementers were found in relation to these influencing factors. The findings contribute to the evidence on implementation quality in schools by advancing knowledge on measuring implementation quality across multiple dimensions and informants successfully. These findings can also inform practitioners of the main influencing factors in schools so that strategies can be developed to optimize implementation quality in the future.

## 1. Introduction

Well-designed evidence-based social and emotional learning (SEL) programs in schools have been shown to be effective in promoting positive outcomes for students [1,2,3,4]. However, variability and fragmentation in implementation quality can lead to inconsistencies in outcome achievement, especially when evidence-based programs are scaled up and delivered outside of controlled research conditions [5,6,7,8]. Research demonstrates that the assessment of implementation quality or rather how well the program delivered aligns with what was intended by developers [9] is essential for determining the validity and overall success of a program [5,6]. In order to accurately interpret the effectiveness of a program, it is necessary to understand how implementation quality varies between sites by answering questions on how much, how well and which aspects of the program were delivered. Systematic monitoring of implementation quality can also help us understand what factors might lead to this variability so that strategies can be developed to optimize the quality of intervention delivery in the future [6,10,11]. 

As awareness of the importance of evaluating implementation quality increases, we also observe a rise in studies reporting on these type of data [3,4,12]. However, review studies that have assessed explicitly the extent of reporting on implementation quality have found that while many studies report monitoring implementation, there are few studies that actually measure and report a quantifiable index of implementation quality [13,14,15]. Thus, a gap remains in adequately assessing the level and extent of variation in implementation quality. In order to assess implementation quality sufficiently, Dane and Schneider [16] recommended the use of a multi-dimensional framework composed of five core dimensions: dosage—quantity of program delivered; fidelity/adherence—how many core components were delivered as prescribed; quality of delivery—how well the facilitator delivers the program; participant responsiveness—how participants respond to or are engaged with an intervention and program differentiation—how unique the program characteristics are compared to other programs. Despite the recognition that multiple dimensions of implementation quality should be used in implementation research, a majority of studies still focus primarily on a singular dimension, most commonly dosage or fidelity/ adherence [6,17]. However, those studies which have assessed other dimensions (e.g., quality of delivery, participant responsiveness, etc.) have found that they could be equally if not more important in influencing program outcomes [17,18,19]. As a way to measure implementation across multiple dimensions, some studies have adopted an approach that employs an a priori index of indicators to calculate a cumulative total implementation index score [20,21,22,23]. For example, the Australian KidsMatter program evaluation combined three dimensions of implementation quality (dosage, adherence/fidelity and quality of delivery) to create a total index score and used this score to categorize schools into high- and low-implementation groups. Groups were then compared in relation to outcomes [21]. Based on the total implementation index score, the findings demonstrated that students in high-implementation schools had significantly greater improvements in SEL skills as well as higher academic outcomes similar to six additional months of schooling. Few other SEL studies however have employed this approach [17]. 

While quantitative data on implementation quality can help determine variability in implementation levels and how this relates to outcome attainment, these type of data are not able to answer why this variability might exist. Qualitative evaluations of implementation answer the “why” by allowing researchers to identify the factors that may contribute to implementation variability. Durlak and Dupre [6] developed an ecological framework to identify five categories for successful implementation (e.g., community level factors, provider characteristics, characteristics of the innovation, organizational capacity factors and factors related to the prevention support system). Similarly, Domitrovich and colleagues [10] presented a multi-level framework of factors that impact on implementation quality in schools at three levels (i) individual-level (e.g., professional characteristics, teacher self-efficacy, perceptions and attitudes towards the program, etc.) (ii) school-level (e.g., resources, school culture and climate, school characteristics, etc.) and (iii) macro-level (e.g., policies, leadership and human capital and university-community partnerships, etc.). The identification and management of these factors is important for better quality implementation.

It is clear that both quantitative and qualitative methods of evaluating implementation are important in implementation research. While a number of studies have assessed implementation using one of these methods, fewer studies have included data on both and even fewer examples of these data being integrated have been reported [24]. Mixed methods designs are favorable in evaluation research because they combine the strengths and balance out the weaknesses, of both quantitative and qualitative research [25]. While quantitative findings may be able to tell us statistically how implementation varies and to what extent this effects outcomes, qualitative data can provide information on why that variability is happening. By integrating the data, factors which are more likely to influence variability in implementation quality can be determined. 

### Current Study 

This study is part of a larger c-RCT study of the MindOut program in post-primary schools in Ireland, which involves three distinct phases. Underpinned by CASEL’s competency framework [26], MindOut is a universal school-based program designed to be delivered by teachers through the SPHE curriculum (Social Personal and Health Education (SPHE) is a mandatory health education curriculum in Irish schools that supports the wellbeing and personal skill development of students [27]) to promote the social and emotional wellbeing of students aged 15–18 years. Additional information on the program and its development can be found in the relevant literature [2,28]. Findings from the c-RCT trial (phase 1) have already been published [29] and revealed significant intervention effects on students’ emotional skills (e.g., coping skills, emotional regulation) and mental health (e.g., reduced stress and depression scores) but found null results in relation to students’ social skills, mental well-being and academic performance outcomes. While the study did show positive findings, it did not account for differences and variability in implementation quality between schools. This information is vital in linking the intervention to the outcomes and determining the program’s validity. Therefore, the aim of this present study (phase 2) is to use a mixed methods approach by employing an implementation quality index to examine the variability in implementation quality for schools implementing the MindOut program and to identify factors that were likely to contribute to this variability through qualitative feedback from participants. The three objectives of this study relate to the three phases of the mixed methods approach:

- To assess variability in implementation quality across four dimensions and determine schools’ overall level of implementation (high/low) using an implementation quality index (quantitative);
- To identify factors that may have influenced the variability in implementation quality as identified by teachers and students (qualitative);
- To examine the relationship between schools’ level of implementation quality and these reported implementation factors (integration).

## 2. Materials and Methods 

### 2.1. Study Context and Design

The original c-RCT study [29] involved 32 schools and 497 students (15–18 years). Participating schools were randomly selected throughout the Republic of Ireland based on criteria that they held the designated disadvantage status (DEIS) by the Department of Education and Skills. Teachers allocated to the intervention group participated in a 1-day training session and were provided with all of the relevant teaching materials to deliver the program. Teachers (n = 17; 93% female) who implemented MindOut and students (n = 280; 53.6% male) that participated in the program were asked to complete quantitative and qualitative measures to assess the process of program implementation during and following the delivery of MindOut. 

This study employed a mixed-methods design analyzing the quantitative and qualitative data independently first, then merging and comparing these data through concurrent triangulation method to enhance the validity and credibility of the findings [30]. In this study, quantitative data indicators were used to determine schools’ quality of implementation across four key implementation dimensions: (i) dosage, (ii) adherence, (iii) quality of delivery, and (iv) participant responsiveness. Following this exercise, the dimension scores were combined to produce a total implementation quality index score, which determined schools’ allocation to either high- or low-implementing groups. The total index score, as employed in previous studies [20,21,22,23], considers all dimensions of implementation quality as equally important and therefore is able to provide a combined score for each school that is inclusive of all these dimensions. 

The qualitative data were used to identify key factors that may have impacted implementation as identified by both teachers and students. Finally, the integration phase of the study allowed for a deeper exploration of the qualitative data in accordance with the schools’ implementation group allocation to determine if the two groups differed in their reporting of influencing factors. 

### 2.2. Measures

All of the process measures were developed specifically for this study to best capture the details of the MindOut program. The teacher and student quantitative measures were piloted prior to the study. Brief descriptions of the quantitative and qualitative measures employed in this study are provided below, and full details are available from the authors on request.

#### 2.2.1. Quantitative Measures: 

**Teacher Weekly Reports:** Teachers in the intervention group were asked to complete weekly questionnaires online following delivery of each of the 12 sessions that included questions on the implementation of the program (e.g., adherence to program content, suitability of the content, student engagement with the session, etc.) The questions from this measure that were used to form the implementation index indicators are outlined in Table S1.

**Student Review Questionnaire:** At the end of program delivery, students from the intervention schools completed a written questionnaire reporting on their experiences of the program (e.g., attendance for specific sessions, teacher’s quality of delivery, their own response/engagement to the program, etc.) Once again, the selected questions from the Student Review Questionnaire that were used to form the implementation index indicators are described in Table S1.

**Classroom Observations:** Classroom observations were conducted by the research team with a random sub-sample of schools during the first and second half of the program (n = 6; 35%). Schools were ranked in random order using Microsoft Excel and the first six schools were visited. Observations were guided by questions on adherence to core components, adaptation, quality of delivery and participant responsiveness. Two researchers visited schools on the same day and completed questionnaires independently followed by a consultation whereby a final score for each item was agreed. Inter-rater reliability for items achieved 83% agreement. As it was not feasible, due to timing and resources, to conduct classroom observations in all intervention schools, this measure was used as a validation tool of the implementation indicators. 

**Implementation Quality Indicators:** Indicators were carefully selected from the Teacher Weekly Reports and Student Review Questionnaire based on their representativeness of one of the four dimensions of implementation [5,6,16]. In this study, “Dosage” refers to the amount of MindOut sessions that were delivered; “Adherence/Fidelity” refers to the extent the program was delivered as intended; “Quality of delivery” refers to how well the teachers delivered the program; and “Participant responsiveness” reflects how students engaged with the program. Indicators that reflected each of the four dimensions were chosen from the teacher and student questionnaires to form an implementation quality index and a scoring system based on these indicators was then created, drawing on the methods used in previous studies [20,21,22,23]. Some of the indicators used single item questions, whereas others were an average score from a number of related questions. Additional details on the indicators can be seen below as well as in Table S1: Details of Implementation Indicators.

**Dosage:** Dosage was measured through 2 indicators. Dosage 1 was measured by asking teachers if they completed the session (yes = 1, no = 0), and this was summed across the 12 sessions. Dosage 2 was measured by asking students to tick (yes = 1; no = 0) if they recalled being present for each of the 12 sessions. Scores were summed across the 12 sessions and students’ scores were averaged within each school. On average, students reported attending 80% of lessons across the 16 schools (range = 41%–95%). Correlations between the two dosage indicators showed that there was a significant relationship between both r = 0.56, p = < 0.05).

**Adherence:** Adherence was measured through 2 indicators from teachers. Adherence 1 was adherence to the key lesson dimensions for each of the 12 sessions based on the Weekly Report data. Different sessions included a different number of items depending on the number of activities (2–4 items; yes = 1, no = 0). The items across all 12 sessions were summed to give a total score. Adherence 2 was measured through a single item, which asked teachers to rate what percentage of each session was delivered (0 = 0%, 6 = 100%). Scores were summed across the twelve sessions and a total mean score was calculated. Correlations were run between the two adherence indictors and demonstrated a significant positive relationship between both (r = 0.81, p = < 0.01).

**Quality of Delivery:** Quality of delivery was measured through 2 indicators. Quality of Delivery 1 assessed students’ rating of their teachers’ delivery of the program (six items, 1= “never” 5= “always”) (e.g., “How often did your teacher show confidence of their own knowledge and skills around each session”). A reliability analysis was conducted which demonstrated a high internal consistency between the six items (α = 0.93). A mean score of the combined items was calculated for each student first, and then these scores were combined for an average school score. Quality of Delivery 2 was a single item asking students to give an overall rating of their teachers’ delivery of the program (1 = “poor”, 10 = “excellent”). Students’ individual scores were averaged for each school to give a mean overall rating. Both of the Quality of Delivery indicators were shown to be highly correlated with each other (r = 0.88, p = < 0.01).

**Participant Responsiveness:** Participant Responsiveness was measured using 4 indicators. Participant Responsiveness 1 was measured based on teachers’ ratings of students’ responses to the activities within each session. An average score was calculated for each individual session, and following this, a total mean score was calculated across all 12 sessions for each school. Participant Responsiveness 2 measured teachers’ perceptions of students’ interaction with each of the 12 sessions based on their interest, skills learned and engagement (3 items; 1 = not at all, 5 = very much) (e.g., “Did the students learn new skills during this session?”). A reliability test was run between the 3 items, which demonstrated that the three items showed high internal consistency (α = 0.98). An average score for each of the three items was calculated first, and a total mean score was then calculated across the 12 sessions. Participant Responsiveness 3 measured students’ response to the program across a number of areas (4 items, 1 = “strongly disagree” 5 = “strongly agree”) (e.g., “The sessions in the program were relevant for me”). A reliability test was run between the 4 items, which demonstrated that the three items showed high internal consistency (α = 0.88). A mean score for each of the four items was calculated for each student, and then, a mean score was calculated by averaging the student scores within each school. Participant Responsiveness 4 used one item to assess students’ overall rating of the program (1 = “very poor” 10 = “excellent”). Students’ individual scores were averaged for each school to give an overall mean. A reliability test was run between the four participant responsiveness indicators and showed an acceptable internal consistency (α = 0.73).

#### 2.2.2. Qualitative Measures: 

**Telephone Interview:** Within 2 weeks of completing the program, teachers (n = 17) were contacted by the researcher via telephone and were asked a series of semi-structured interview questions in relation to their overall experience of implementing the program (e.g. what worked well, challenges, student response, future implementation/sustainability, etc.).

**Student Participatory-based Workshops:** Participatory-based workshops were conducted with a random sub-sample of schools (n = 5). These schools were selected from the randomized list used for the classroom observations, and the next 5 schools on the list (#7-11) were contacted for these workshops. The workshops were based on the methodology used in other studies [31,32] and included interactive student-centered approaches including voting games, group work, and post-its exploring students’ views on their experience of the MindOut program (e.g., what they liked, what they did not like, what improvements could be made, etc.). 

**Student Review Questionnaire:** The Student Review Questionnaire also contained open-ended questions that gathered qualitative information on the students’ program experience (e.g., “What aspects of the MindOut program do you think worked best?” and “What suggestions do you have for improving the MindOut program?”).

### 2.3. Analysis

#### 2.3.1. Quantitative Analysis

All of the indicator scores were aggregated at the school level and then converted into a percent score, which allowed for the indicator scores within each dimension to be averaged for a total dimension score. A similar process has been carried out in other implementation studies [33,34]. Internal reliability checks and correlations were run between indicators within the same dimension. A reliability analysis was also carried out on all four total dimension scores and found a high internal consistency for the items (α = 0.86). The four total dimension scores (dosage, adherence, quality of delivery and participant responsiveness) were averaged for a Total Implementation Quality score.

Total Implementation Quality = (Total Dosage + Total Adherence + Total Quality of Delivery + Total Participant Responsiveness) / 4

There was no weighting of items, therefore, all dimensions contributed equally to the total implementation quality score. Similar methods of combining indicators across dimensions to produce a total index score are found in other studies [20,21,22,23]. Classroom observations were used to validate the self-report measures by comparing with indicator scores of relevant dimensions to check for compatibility. In order to classify schools into their implementation groupings, the visual binning procedure was employed using SPSS as used by Dix and colleagues [21]. Binning was performed by applying cut-points at the mean and ±1 standard deviation, resulting in four binned categories (1 = low, 2 = moderately low, 3 = moderately high, 4 = high). Based on these binned scores, schools were allocated to their implementation level group. Using this procedure, the distribution of schools across the categories was examined, and in view of the small number of schools in certain categories, an allocation into two overall categories of high and low was made. Schools that received a binned score of “1 or 2” were considered low implementers and those that received a score of “3 or 4” were deemed high implementers. The visual binning procedure was completed for each of the four dimensions as well as for the total implementation quality score.

#### 2.3.2. Qualitative Analysis

All teacher telephone interviews were audio-recorded and transcribed verbatim via a transcription service. Thematic analysis [35] was used to analyze the teacher and student data. Transcripts were read several times, and meaningful units of text were highlighted, summarized and coded. A subset of transcripts was double coded by an independent coder to check for inter-rater reliability. All relevant data from the student measures were extracted and coded. All data were managed in Microsoft Word. For both the teacher and student data, codes discussing similar ideas or issues were grouped into sub-themes. Following this, sub-themes were further grouped into overarching themes and comparisons between the teacher and student data were made.

#### 2.3.3. Integration

Integration of quantitative and qualitative data occurred at an interpretation level using the concurrent triangulation method [30]. Each theme identified in the qualitative data was further analyzed according to the schools’ group allocation, which was determined by the quantitative data. Using the themes to guide the process, the data were analyzed, and similarities and differences between the two groups were identified and recorded in a matrix to facilitate the comparison of the findings.

## 3. Results

### 3.1. Quantitative 

#### 3.1.1. Demographics 

A total of 16 schools (n = 280 students) were included in this study. (One school completed less than 50% of the Teacher Weekly Reports and, therefore, could not be included in the analysis or assigned to an implementation group.) A majority of schools (63%) chose to deliver the program with a 5th year group instead of a Transition Year (TY) class. (Transition Year (TY) is a one-year optional program that exists between the Junior Certificate program (3rd year; 13–15 yrs.) and the Leaving Certificate program (6th year; 16–18yrs). Transition Year is a less structured year which gives students more space to learn, mature and develop without the presence of exam pressures [36].) The average class size for participating schools was 17.06 (SD = 5.16; range = 8–29). 

#### 3.1.2. Dose

Twelve of the sixteen schools implemented the MindOut program in its entirety. Of the four schools that did not complete the program, two delivered less than 60% of the program. Students reported attending 80% of lessons on average across the 16 schools (range = 41%–95%). Based on the visual binning procedure, six schools were classified as low implementers for dosage. 

#### 3.1.3. Adherence

On average, teachers reported delivering 71% of the key activities and adhered to 82% of the sessions. Seven schools were identified as low implementers for adherence using the visual binning procedure. Classroom observation scores from the specific sessions the researchers observed were used to validate the teachers’ self-report measures from the same session. Based on the total number of delivered activities on the day, all six schools received a classroom delivery score that was within 10% of the teachers’ adherence score based on their reporting of delivered activities.

#### 3.1.4. Quality of Delivery

The average rating given by students for teachers’ quality of delivery was 76% (range = 57%–93%). Eight schools were identified as high implementers for quality of delivery based on visual binning. Once again, classroom observations scores were used to check validity of the self-report data. All six schools that participated in classroom observations had classroom observation scores that were within 10% of the total quality of delivery score. Given that classroom observation scores for quality of delivery were based on one session only and indicator score reflected quality of delivery over the course of the program, slight differences in these scores could be expected.

#### 3.1.5. Participant Responsiveness 

Based on teacher reports, an average rating of students’ interaction with the sessions was calculated across the 12 sessions for each school, and the mean score across all schools was 77% (range = 59%–95%). A total of 75% of students rated the program at 7 or above on a 10-point scale. In accordance with the visual binning procedure, eight schools were identified as high implementers. Classroom observations scores were used to check for reliability. All six schools that participated in classroom observations had total participant response scores that were within 10% of the classroom observation scores. Again, as classroom observation scores for participant responsiveness were based on a single session only whereas the indicator scores were based on the entire program, it is likely that there would be slight differences between the scores.

The means and ranges for schools across all dimensions can be found below (Table 1). The visual binned scores were compared across all four dimensions to determine how frequently each school scored in each group. Three schools ranked high or moderately high in all four dimensions, and two schools ranked low or moderately low in all four dimensions. All other schools (n = 11) ranked high in some dimensions but low in others (see Table S2: Visual Binning Scores for schools across dimensions and total implementation quality).

#### 3.1.6. Total Implementation Quality 

Based on the visual binning procedure for Total Implementation Quality, two schools were in the low group (1), six in moderately-low (2), six in moderately-high (3) and two in the high group (4). After combining the low/moderately low schools into one group (low implementation) and the high/moderately high schools into the other group (high implementation), eight schools were assigned to both of these groups. The schools that were identified as high implementers for Total Implementation Quality all ranked in the “high” group for at least three of the four dimensions. In comparison, all of the schools identified as low implementing scored in the “high” group in two or less dimensions. The visual binning scores for each of the individual dimensions as well as for Total Implementation Quality can be found in Table S2: Visual Binning Scores for schools across dimensions and total implementation quality.

### 3.2. Qualitative

**Figure 1:**
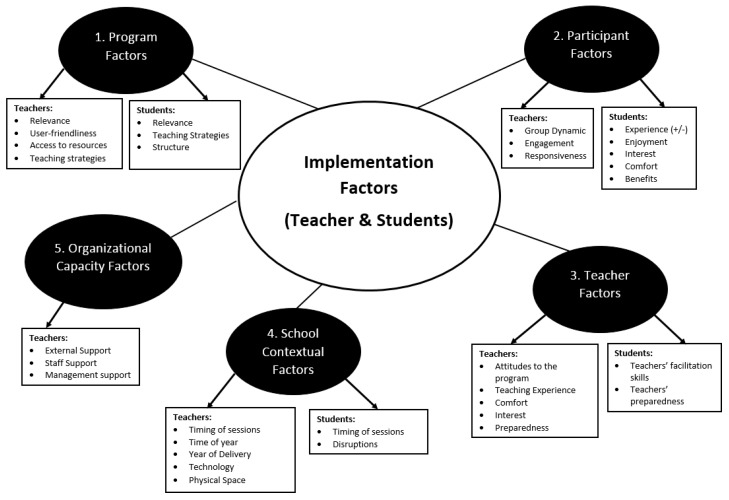
Implementation factors and themes: Teacher and student data.

Five themes were identified from the teacher Telephone Interview data: (i) Program factors, (ii) Participant factors, (iii) Teacher factors, (iv) School contextual factors and (v) Organizational capacity factors. The first four themes were also identified through the Student Review Questionnaire and Participatory Workshop data. The fifth theme was only identified as an influencing factor by teachers. The themes and sub-themes for both the teacher and student data can be found in Figure 1. 

#### 3.2.1. Program Factors

Program factors included the program’s relevance, accessible resources, user-friendliness and teaching strategies. Most teachers felt that the program was age-appropriate for senior students, “I think the fact that all of the content was aimed at that age group, and it was relevant to them. I feel that it went down a treat” with a few teachers suggesting some sessions might suit a younger group. Teachers also felt that the content within the program was culturally relevant for Irish students and thought the lessons were current and timely, “There are so many good practices in here and it’s also culturally ... you know it’s pitched in the right way for the Irish culture.” There was a general consensus from teachers that the resources included useful material that was well laid out and easy to access. Program factors discussed by students included relevance, variety of teaching strategies and the program structure. A majority of students felt that the program was relevant for their age and generation, but a few students could not relate to the content. Students commented on the teaching strategies and said they preferred the interactive teaching strategies, group work and videos, “it was a good way of learning about mental health and made it interesting by using videos and activities so it wasn’t as boring and fully theory”.

#### 3.2.2. Participant Factors

Participant factors identified by teachers included group dynamic as well as students’ engagement and response to the program. In relation to group dynamic, some teachers felt that the program was easy to implement because of their group, “I suppose I was very lucky with the particular group I had. They were very willing to be involved.” Other teachers felt the group dynamic negatively affected implementation “Their resilience would be very low…they would be weaker students so they wouldn’t necessarily have the vocabulary to ... or the confidence to discuss emotional issues.” There were mixed reports on students’ responsiveness and engagement in the program with some teachers saying their students responded well and others saying the students were disengaged and uninterested. Participant factors discussed by students included their reported experiences of the program, experienced benefits of the program, as well as their enjoyment and interest with the sessions. The majority of students had positive things to say, “it is excellent for all people”, but a few students had negative feelings towards the program, “I didn’t enjoy it or think it was worthwhile”. A majority of students commented on how the program benefitted them, stating that it was helpful and providing examples of specific skills they had developed. In terms of enjoyment and interest with the program, again, the results were mixed with many students finding the program enjoyable and interesting and others commenting that parts were boring. 

#### 3.2.3. Teacher Factors

Teacher factors included teachers’ attitudes to the program, their comfort and interest in the content, their preparedness for the lesson as well as their own teaching experience and previous training. In general, teachers were positive towards the program commenting that it was enjoyable to deliver and that the content was valuable. Many teachers stated the importance of being trained in SPHE before delivering MindOut and being comfortable and interested in the content of the program, “It’s important that the teacher ... is interested or passionate about this whole area of mental health because then they’ll be more confident in delivering it and hopefully then the students can take more from it then.” Teacher factors for students were related to teachers’ facilitation skills and preparedness. Some students felt their teacher delivered the program poorly and should have been better prepared, “my teacher wasn’t engaging with us properly and didn’t have some things ready for the class”. 

#### 3.2.4. School Contextual Factors

School contextual factors described by teachers included the timing of the sessions, the time of year the program is delivered, year of delivery, physical space and technology. Many teachers commented on issues with the timing of the program in regard to completing the sessions during the 35 min class period. These teachers commented on how this impacted the delivery such as cutting activities from the session, inability to complete activities well and failing to complete sessions altogether, “…and while I did get them done they weren’t done you know as well as they should have been”. Teachers also highlighted the importance of starting the program early in the academic year as they get busier and less likely to find time to deliver as the year progresses. There were mixed opinions on which year group was best suited to receive the program. Some schools favored TY due to the lack of exam pressures and more provision to implement, while and other teachers felt that TY was too busy and students were more likely to be absent compared to 5th year, “…a lot of the time there were people missing because it’s TY and they are busy doing all sorts of different things in the school.” School contextual factors mentioned by students included the lack of time for the sessions and classroom disruptions. Some students stated that they felt program sessions were rushed, “we were very rushed for time making it slightly stressful”. A few students discussed how their peers’ disengagement (e.g., messing, lack of attention, etc.) interfered with completing the sessions on time. 

#### 3.2.5. Organizational Capacity Factors

Organizational capacity factors included external support, staff support, and support from management. Teachers felt that support from other staff in terms of awareness and helping to deliver the program would be important for sustainability of the program. Some teachers were concerned that other staff members and management did not value the program, “I do think it would have been easier if management had given me that little bit more support… I just didn’t have it.” In terms of external support, teachers expressed that they would like to receive updates from Health Promotion Officers in terms of relevant material and would benefit from meeting with other teachers delivering the program to learn from their experiences and feel more supported. Students did not refer to any factors for this theme. 

### 3.3. Integration 

#### 3.3.1. School Profile 

In comparing the demographic data between the two groups, there was a noticeable difference in average class sizes with the high implementers reporting larger group sizes (M = 19.7 SD = 5.34) compared to the low implementers (M = 14.5 SD = 3.67). In terms of year of delivery, a majority of the high-implementing group (75%) delivered the program to 5th years and a majority of the low-implementation group (63%) delivered the program to TY groups. 

The themes from the qualitative data were further analyzed in accordance with school implementation level grouping to highlight the differences and similarities between the two groups. Further details of this analysis can be found in Table S3: Similarities and differences between high- and low-implementing groups (teacher and student data). 

#### 3.3.2. High-Implementation Group

A number of factors were identified in the data from the high-implementation schools which were unique to this group. In terms of the participant characteristics, the high-implementation group tended to speak more positively about the dynamic of their group (e.g., talkative, engaged, cooperative, etc.) and were more likely to indicate higher student engagement and positive responses to the program. For student data, high implementers were more likely to discuss benefits of the program by providing examples of specific SEL skills they had developed. Whilst there were mixed views for both groups in terms of levels of interest in the program, in general, the high-implementation students made more positive comments. For teacher characteristics, high-implementing teachers demonstrated more positive attitudes when discussing their experience of the program. For organizational factors, high-implementing schools reported a need for more external support from Health Promotion Officers and agencies to continue to build their skills around SEL and mental health. 

#### 3.3.3. Low-Implementation Group 

Likewise, a number of specific factors concerning the low-implementation schools also emerged from the data. For participant characteristics, the teachers in the low-implementation group spoke more negatively about the dynamic of their group (e.g., difficult, high-need, issues, low-emotional literacy, etc.) and were more likely to comment on student disengagement, lack of interest and negative responses. Similarly, the students in the low-implementation group reported more negative experiences of the program and tended to speak in more general terms when discussing the perceived benefits of the program (e.g., “helpful”, “useful”, “learned new things”, etc.). In terms of teacher characteristics, students in the low-implementation group reported negative comments about their teachers’ facilitation skills, whereas the high-implementation students did not make any reference to this. In relation to school contextual factors, the low-implementation group commented on issues with delivering the program to a TY group (e.g., missing classes, less consistency week-to-week, etc.), whereas the high implementers, a majority (75%) of which delivered the program to 5th years, did not discuss any similar issues. Students in the low-implementation group reported experiencing more issues with classroom disruptions and peer disengagement. Finally, in terms of the organizational capacity factors, the low-implementation teachers group expressed their desire for more support from school management for delivering the program.

#### 3.3.4. Similarities 

There were no differences between the two groups for “Program factors” based on teacher feedback. Both groups reported that the program dealt with current issues, was relevant and user-friendly. For teacher characteristics, both groups suggested that the teachers’ own teaching experience, as well as their comfort and interest in the program, were essential for implementation. In relation to “School contextual factors”, both groups expressed the importance of introducing the program early in the year and having it completed before the spring term. Both groups agreed that the timing of the sessions (e.g., completing the session in a 35-minute class period) was one of the most difficult challenges faced during implementation. Students of both groups reported finding the program long and suggested shortening some of the sessions. For “Organizational capacity factors”, teachers in both groups reported that they would have liked more support from other staff within the school. 

## 4. Discussion

This study set out to assess the variability in implementation quality of schools delivering the MindOut program and to investigate the factors that most likely contributed to this variability, particularly in relation to schools’ implementation level. These aims were achieved through a mixed methods approach assessing quantitative and qualitative data from teachers and students and integrating these to form a clearer picture of implementation quality in schools. A major finding from this analysis was that variability in implementation quality existed both between and within schools, which is in line with other research [21,22,23]. In particular, the findings from this study show that there was considerable variability in implementation quality between schools, even when the training and resources they received were identical. While some schools rated rather high in implementation quality, other schools, delivering the same program, scored relatively low (range 55%–92%). Given that programs can lose their effectiveness when they are implemented poorly, this is an important finding within the context of the original c-RCT study, which did not account for implementation level differences. The other interesting finding from this study is that variability in implementation also occurred within schools. Noticeable inconsistencies were found within schools in relation to implementation quality across different dimensions (e.g., dosage, fidelity/adherence, quality of delivery, participant responsiveness). Of the 16 schools in this study, seven consistently scored either high (n = 4) or low (n = 3) across all four dimensions. All other schools varied, scoring high in certain dimensions and low in others. This finding justifies the importance of measuring implementation quality across several dimensions, as no single dimension is reflective of implementation quality as a whole. Lastly, when comparing the results of the teacher and student data within each dimension, these data were not correlated. This suggests that data from teachers and students provide different perspectives on important aspects of implementation. Therefore, there is a need to gather data on implementation from multiple informants to ensure all stakeholders’ perspectives are considered. 

This study also examined factors that affected implementation as identified by both teachers and students. While the sample included a range of mixed gender schools, the majority of the teachers involved were female. It is therefore unclear to what extent these findings on teachers’ perspectives would also apply to male teachers. Consistent with the findings of previous studies [6,10,11], our analysis revealed a range of factors that contribute to variability in implementation. Clear links can be made between the factors within this study and those identified in Durlak and Dupre’s [6] and Domitrovich’s et al. [10] models. “Community-level” [6] and “macro-level factors” [10] were not however identified within the teacher or student data in this study. In the current study, participants were asked about their experiences of the program more generally, and an inductive thematic approach was used, whereas in other studies, the frameworks described above [6,10] have been used to guide the interviews and analysis process through a deductive approach. Therefore, within the context of this study, it is likely that individual- and school-level factors were thought to be most important for implementation. In this study, participant factors (e.g., group dynamic, engagement, responsiveness, etc.) were considered by both teachers and students as important factors for implementation. Though these factors are not specifically referenced in the frameworks discussed above, a few studies have acknowledged the importance of participant-type factors on implementation such as participants’ engagement, attitudes and motivation [37,38,39,40]. These participant-type factors not only can have either a direct positive or negative impact on teachers’ willingness and motivation to deliver the program well but can also be key contributors to the overall implementation quality of the program [37,38,40,41].

A unique aspect of the present study is that it also examined whether or not high- and low-implementation groups differed in their reporting of implementation factors. The analysis found that schools in the high-implementation group were more likely to have larger class sizes in comparison to the low-implementation group, which contradicts previous studies indicating that increased class sizes lead to decreases in quality implementation [42,43]. It is possible that schools with smaller class sizes may have pre-selected a group of higher-need students to which deliver the program that could have affected the implementation quality (e.g., more disruptions, lower attendance, lower engagement, more complex behavioral issues, etc.). Poorer classroom behaviors, misconduct, peer conflict and classroom management have all been identified as predictors of weaker implementation [10,42,43,44]. It is also possible that the MindOut program is easier to implement with larger class sizes due to the interactive nature of the activities, which require a certain number of students to work effectively. The groups also differed in terms of the year group they selected to deliver the program. Although all schools were given the choice to deliver the program to either a TY class or 5th year class, a larger number of low-implementing schools selected the former whereas a majority of high implementation schools selected the latter. The low implementers commented on difficulties they had faced delivering the program to TY students due to class interruptions, lack of structure and poor attendance; the high implementers, however, did not reference these types of issues. Teachers in high-implementation schools were also more likely to make positive statements about their group dynamic and their students’ engagement whereas low implementers commented more negatively about these aspects. Based on these findings, the importance of group characteristics for successful implementation is apparent. Schools deciding to implement this type of program need to consider the characteristics of their group in planning delivery. Like other SEL programs, MindOut is a universal program intended to be delivered to all students in the classroom ranging in needs and abilities. Additionally, schools should consider a group who can participate in the program consecutively on a weekly basis. Interruptions in implementation lead to disjointed programs that are likely to have lower participant-engagement and reduce their overall effectiveness.

In terms of “Teacher factors”, teachers of higher-implementing schools demonstrated more positive attitudes towards the program compared to low implementers. Perceived value of the program, acceptance of the intervention and its perceived effectiveness are all factors that lead to stronger implementation [10]. Therefore, it is not surprising teachers of high-implementing schools were more positive when discussing the program and its benefits. Additionally, students of low-implementing schools commented on their teachers’ poor facilitation skills where students in the high-implementing group did not. Poor facilitation skills can be directly linked to the dimension “quality of delivery”. Further, when a facilitator lacks in areas of quality of delivery (e.g., enthusiasm, engaging, control, preparation, etc.), it is likely to have an impact on how students respond to the program (participant response) and thus the overall quality of implementation. A final key difference between the two groups was in terms of “organizational factors”. While both groups expressed the need for more support from other teachers and staff members, the low implementers were more likely to say they wanted more support from management. This is an important finding as it highlights the need for interventions like MindOut to be a priority and supported by management to ensure stronger implementation quality. One common issue raised by both the high-and the low-implementation groups was the pressure of timing when delivering the sessions. Teachers and students expressed that sometimes sessions felt rushed or there was not enough time in the class period to get through all aspects of the session with quality delivery. Teachers and students were asked how this could be improved, and the main suggestions included lengthening the program to be delivered over more weeks, working with management to timetable the program into double-class periods and reducing some of the content. Based on this feedback, modifications were made to the final program. In order to ensure high-quality implementation and sustainability of school-based programs, it is vital that the timing of the program and sessions be considered carefully.

### 4.1. Strengths and Limitations

This study employed a mixed-methods design using the strengths of both quantitative and qualitative research. Unlike a majority of implementation studies that focus primarily on data from program implementers only, this study assessed implementation based on feedback from both teachers and students. While feedback from the implementer is important, students provide a different perspective and additional data, particularly in relation to the quality of delivery and participant responsiveness. A major limitation of this study is that implementation was assessed primarily using self-report data, which is often criticized for being biased and over-estimating implementation practices [45]. Though self-report data has its limitations, due to time and resource constraints, it was deemed the most feasible method for this study. To deal with the likelihood of overestimating implementation in the self-report measures, we used data from teachers and students to create a combined average for implementation quality. We also used observer data to validate the self-report measures. Another limitation of this study is that the implementation indicators used were not validated, as these were developed specifically to assess implementation directly related to the MindOut program. However, the study could have been strengthened by using implementation measures with well-established psychometric properties. 

### 4.2. Implications and Future Directions

This study demonstrated that variability in implementation existed both between schools as well as within schools, across different dimensions. This highlights the need for researchers to systematically assess implementation quality in evaluation studies to ensure benefits of a program are not lost due to poor implementation. This finding also provides a rationale for evaluating implementation across several dimensions given that so much variability can be seen within schools. Future research assessing how implementation variability influences outcome achievement will be important within the context of this program evaluation. The next phase of this study (phase 3) will explore this, by assessing how variability in implementation quality impacts on students’ outcomes. This study also provided a rich narrative on what factors may be contributing to the variability in implementation quality between schools. More specifically, these data highlight the factors that are more likely to be associated with high- and low-implementing schools. These findings are especially important for program developers and practitioners as it allows them to embed strategies into the planning and delivery stages of implementation (e.g., school-readiness, management buy-in, training, ongoing support for delivery, etc.) in an effort to optimize quality of implementation, which will lead to better outcomes for participants. 

## 5. Conclusions

This study demonstrated an effective way of assessing implementation quality of a school-based program by scoring schools on a range of indicators and combining these scores to produce a total implementation score for each school. Based on the total implementation quality score, eight schools were identified as high implementers, and another eight were low implementers. Not all schools scored consistently high or low across all four dimensions. This finding highlights that variability in implementation quality not only existed between schools but within schools as well. This study also identified the factors that are likely to contribute to the variability in implementation quality as defined by teachers and students. Finally, this study was able to determine if certain factors were more likely to modify implementation quality when comparing high- and low-implementation groups. These findings contribute to the evidence on implementation quality in schools by advancing knowledge on measuring and assessing implementation quality across multiple dimensions successfully. The findings also help to inform practitioners of the factors that impact implementation variability so that strategies can be developed to mitigate this variance in the future. The findings from this study will help to inform the next stage of the study, which will examine how variable levels of implementation quality relate to program outcomes.

## Figures and Tables

**Table 1:** Means and ranges of schools’ total dimension scores.

Dimension	Mean	Range
Total Dosage	86%	46%–98%
Total Adherence	77%	44%–100%
Total Quality of Delivery	76%	57%–93%
Total Participant Responsiveness	75%	62%–89%
Total Implementation Quality	79%	53%–92%

## Supplementary Materials

The following are available online at https://www.mdpi.com/1660-4601/17/9/3249/s1. Table S1: Details of Implementation Indicators, Table S2: Visual binning (VB) scores for schools across dimensions and total implementation quality, Table S3: Similarities and differences between high- and low-implementing groups (Teacher and Student data).
