# Supplementary material for: Evaluating the Implementation Quality of a Social and Emotional Learning Program: A Mixed Methods Approach
Source: Int J Environ Res Public Health. 2020 May 7;17(9):3249. doi: 10.3390/ijerph17093249 (PMC7246810; doi:10.3390/ijerph17093249)
Supplement: Supplementary file 1 [file ijerph-17-03249-s001.pdf]

Table S1: Details of Implementation Indicators

Fidelity Measure	Instrument	Indicator	Question	Rating Scale	Scoring
**Dosage**					
1	Teacher Weekly Report	Delivery of each session	• Did you deliver this session?	No=0<br>Yes=1	• Summed score across 12 sessions.<br>• Convert to percent score
2	Student Review Questionnaire	Student attendance for each session	• Tick all the sessions you remember being present for.	No=0<br>Yes=1	• Summed score across 12 sessions.<br>• Average score for students within each school.<br>• Convert to percent score
**Adherence**					
1	Teacher Weekly Report	Key lesson activities implemented (3 items)	• Were you able to complete activity 1 in the time?<br>• Were you able to complete activity 2 in the time?<br>• Did you play the recommended video?	No=0<br>Yes=1	• Summed score for each session.<br>• Summed score across 12 sessions.<br>• Convert to percent score
2	Teacher Weekly Report	Teacher rated adherence to each session (1 item)	• What percentage of the session did you complete?	0% = 1<br>100% = 6	• Average score across 12 sessions.<br>• Convert to percent score
**Quality of Delivery**					
1	Student Review Questionnaire	Student ratings of aspects of teacher quality of delivery (6 items)	• Please rate how often your teacher did the following during the delivery of MindOut:<br>• Was confident in their own knowledge and skills around each session.<br>• Was enthusiastic (passionate) when delivering the sessions.<br>• Made critical or negative remarks about the students during the sessions (Reverse)	Never = 1<br>Always = 5	• Average score for all 6 questions for each student.<br>• Average score for students within each school.<br>• Convert to percent score

			• Showed appreciation when students shared comments and participated<br>• Kept students engaged and interested in the session.<br>• Seemed well-prepared and organized for the sessions.		
2	Student Review Questionnaire	Individual student rating teacher quality of delivery overall (1 item)	• Please rate from 1 (poor) to 10 (excellent) how well your teacher delivered the MindOut program.	Poor = 1<br>Excellent = 10	• Average score for students within each school.<br>• Convert to percent score
**Participant Responsiveness**					
1	Teacher Weekly Report	Teacher rating of students response to key lesson activities (3 items)	• How did the students respond to the first activity?<br>• How did the students respond to the second activity?<br>• How did the students respond to the video(s)?	Very Poorly=1<br>Very Well = 5	• Average score for all 3 questions within each session.<br>• Average score across 12 sessions.<br>• Convert to percentage
2	Teacher Weekly Report	Teacher rating of students' interaction with the session (1 item)	• Did the students show interest in this session?<br>• Did the students learn new skills during this session?<br>• Did the students engage/participate in the activities and discussions?	Not at all=1<br>Very much = 5	• Average score for all 3 questions within each school across 12 sessions.<br>• Convert to percentage
3	Student Review Questionnaire	Students' ratings of their interaction with the program (4 items)	• The sessions in the program were relevant for me.<br>• The sessions in the program were useful for helping to deal with situations.<br>• The content of the program sessions was easy to understand.<br>• The sessions in the program were interesting.	Not at all=1<br>Very much = 5	• Average score for all 4 questions for each student.<br>• Average score for students within each school.<br>• Convert to percentage
4	Student Review Questionnaire	Students' rating of the program overall (1 item)	• How would you rate the MindOut program overall?	Poor = 1<br>Excellent = 10	• Average score for students within each school.<br>• Convert to percent score

Table S2: Visual binning (VB) scores for schools across dimensions and total implementation quality

School ID	**Dosage Total VB score**	**Adherence Total VB score**	**Quality of Delivery Total VB score**	**Participant Responsiveness Total VB score**	**Total Implementation Quality VB score**	**Total Implementation Quality Group**
#1	3	2	2	1	2	Low
#2	3	3	4	2	3	High
#3	1	1	2	1	1	Low
#4	3	3	1	1	2	Low
#5	1	1	1	1	1	Low
#6	2	2	2	2	2	Low
#7	3	4	3	4	4	High
#8	3	4	3	3	3	High
#9	3	3	3	3	3	High
#10	2	2	1	3	2	Low
#11	2	3	2	2	2	Low
#12	2	3	3	3	3	High
#13	3	3	4	4	4	High
#14	3	2	4	3	3	High
#15	3	2	2	2	2	Low
#16	3	4	2	3	3	High

[a] VB = Visual Binning Score, [b] Low =1; Moderately Low=2; Moderately High=3; High=4, [c] Total Implementation Quality Group: Low =8; High=8

Table S3: Similarities and differences between high- and low-implementing groups (Teacher and Student data).

Themes	Informant	High-implementation group (n=8)	Both Groups	Low-implementation group (n= 8)
**1. Program Content Factors**	**Teacher**		Felt the program was relevant for students.<br><br>Commented on the program's user-friendliness and easy accessibility to resources.<br><br>Praised the variety of teaching strategies, particularly the interactive elements.	
	**Student**		Thought that some sessions in the program were too long or felt rushed.	Reported that they experienced disruptions during the sessions and/or peer disengagement.
2. **Participant Characteristics**	**Teacher**	More positive comments when discussing group dynamic.<br><br>More likely to discuss students' engagement and response to the program in a positive light.		More issues with the group dynamic (high-need, difficult, low emotional literacy, low resilience etc.)<br><br>More negative comments in terms of student engagement and response to program.
	**Student**	Reported more benefits from the program in terms of helpfulness.<br><br>Provided specific examples of how the program impacted on their learning and skill development.	Found some parts of the program boring.<br><br>Found the program interesting.	More likely to report negative experiences with the program.<br><br>Less likely to report the program benefitting them and did not provide examples of skill development.

		More likely to report enjoyment of the program and discuss positive experiences.		
3. **Teacher Characteristics**	**Teacher**	More likely to demonstrate positive attitudes towards program.	Felt that comfort, interest and teaching background were important for implementation.<br><br>Agreed the training was good.	
	**Student**			Commented on their teachers' poor delivery of the program.
4. **School Contextual Factors**	**Teacher**		Identified 'timing' as a major issue in implementing the program.<br><br>Felt delivering the program early in the year would make implementation easier.	Discussed issues delivering the program to TY students.<br><br>Faced issues with accessing technology.
	**Student**		Thought that some sessions in the program were too long or felt rushed.	Reported that they experienced disruptions during the sessions and/or peer disengagement.
5. **Organizational Factors**	**Teacher**	Expressed a desire to receive external support in terms of updates from Health Promotion Officers and support groups for teachers implementing.	Felt other staff didn't value the program and that they would like more support from their colleagues.	Would have liked more support at the management level.<br><br>Felt they could be supported by having an external person visiting the school to deliver the sessions.
